# Development and external validation of a prognosis model to predict outcomes after curative resection of early-stage intrahepatic cholangiocarcinoma

**DOI:** 10.3389/fsurg.2023.1102871

**Published:** 2023-03-08

**Authors:** Jianping Wang, Manling Huang, Jingxian Shen, Bin Li, Yanqing Wu, Wenxuan Xie, Han Xiao, Li Tan

**Affiliations:** ^1^ Department of Gastroenterology and Hepatology, The First Affiliated Hospital, Sun Yat-Sen University, Guangzhou, China; ^2^ Department of Oncology, Cancer Center, The First Affiliated Hospital, Sun Yat-Sen University, Guangzhou, China; ^3^ Institute of Precision Medicine, The First Affiliated Hospital, Sun Yat-Sen University, Guangzhou, China; ^4^ Department of Medical Imaging, State Key Laboratory of Oncology in Southern China, Sun Yat-Sen University Cancer Center, Guangzhou, China; ^5^ Clinical Trials Unit, The First Affiliated Hospital, Sun Yat-Sen University, Guangzhou, China; ^6^ Center of Hepato-Pancreato-Biliary Surgery, The First Affiliated Hospital, Sun Yat-Sen University, Guangzhou, China; ^7^ Department of Medical Ultrasonics, The First Affiliated Hospital, Sun Yat-Sen University, Guangzhou, China

**Keywords:** intrahepatic cholangiocarcinoma, resection, prognosis, risk, nomogram

## Abstract

**Background:**

Early-stage intrahepatic cholangiocarcinoma (ESICC) with curative resection and lymph node-negative still has the risk of poor prognosis, and there lacks prognosis-assessing tools for these patients. The objective of this study was to develop a prognosis model to predict outcomes and identify risk stratification for ESICC after resection.

**Methods:**

Totally 263 patients with ESICC after hepatectomy from January 2012 to January 2022 were analyzed. Clinicopathological factors were selected using multivariable Cox regression analysis and a prognosis model was developed. The performance of the model was evaluated by concordance index (C-index), calibration plots, decision curves analysis (DCA), and net reclassification index (NRI). Kaplan-Meier curves were analyzed for risk stratification of overall survival (OS) and recurrence-free survival (RFS) based on the prognosis model.

**Results:**

The clinicopathological features that were independently associated with OS of ESICC included carbohydrate antigen19-9, carcinoembryonic antigen, tumor size, tumor differentiation, and T stage. The prognosis model based on these prognostic factors demonstrated excellent discriminatory performance in both derivation cohort (C-index, 0.71) and external validation cohort (C-index, 0.78), which outperformed the TNM staging system (C-index, 0.59) and individual prognostic factors (all C-index < 0.7). Calibration plots, DCA and NRI also showed superior predictive performance. According to the risk for survival, the model stratified patients into low risk (median OS, 66.6 months; median RFS, 24.3 months) and high risk (median OS, 24.0 months; median RFS, 6.4 months) (*P* *<* 0.001).

**Conclusions:**

Our prognosis model can robustly predict the outcomes of ESICC after curative resection and provide precise evaluation on prognosis risk, facilitating clinicians to develop individualized postoperative treatment options.

## Introduction

The global incidence and mortality rates of intrahepatic cholangiocarcinoma (ICC) have been consistently increasing in recent decades, with the 5-year overall survival (OS) less than 10% (1, 2). Radical liver resection remains the standard of care and the only promising curative treatment option for ICC. But the long-term outcome after resection is still dismal, with the 5-year OS < 40% and the initial recurrence in 60%–70% (3, 4). Positive surgical margins and regional lymph node metastases are typically recognized as critical factors for poor prognosis after resection (3). As a result, current guidelines generally recommend postoperative adjuvant therapy for these patients (5). However, for early-stage ICC (ESICC), who are completely resected (R0) and lymph node-negative, some patients still experience recurrence after surgery, with the median recurrence-free survival (RFS) less than 40 months (6–8). And in such patients, the postoperative treatment options recommended by the National Comprehensive Cancer Network (NCCN) are arbitrary (9). Several retrospective studies have shown that administration of postoperative adjuvant therapy for ESICC patients may prolong survival (10, 11), while other studies have failed to reach consistent conclusions on the effectiveness and role of adjuvant therapy for ESICC (12, 13). Consequently, it highlights the stratification of ESICC in terms of risk of postoperative recurrence and survival, which will bring immense value for assisting personalized treatment options.

Each tumor has unique tumor biological characteristics, which are widely associated with malignant progression, therapeutic response, and diverse prognosis (14). In current NCCN guidelines, postoperative treatment choices for ICC are based only on surgical margins and regional lymph node status (9), which now appear to be insufficient for assessing the prognosis of ESICC. Since ESICC is characterized with R0 resection and negative lymph nodes, the 5-year OS is as poor as 30%–50% (6–8). On the other hand, few is known about the role of other tumor characteristics besides margin and lymph node, which may be the potential determinants of survival for ESICC. Therefore, for ESICC patients, a robust prognosis model comprehensively incorporating high-risk tumor features is needed to allow clinicians and patients to make well-informed decisions regarding adjuvant therapy and postoperative cancer surveillance. Emerging evidence now indicates that clinicopathologic features have the potential to reflect tumor growth and invasion ability, and have increasingly been integrated into prognosis evaluation to identify actionable prognostic factors as early as possible (15–17). To our knowledge, no studies have so far used clinicopathologic features to develop predictive prognosis models in patients with ESICC for risk stratification and guiding cancer care.

This study aims to find clinicopathological variables associated with the prognosis of ESICC, and to develop and validate a prognosis model for evaluating the survival outcomes of ESICC patients, in order to accurately stratify survival risk and provide assistance in clinical decision-making for individual patients.

## Methods

### Study patients

This is a retrospective two-center clinical cohort study based on a dataset of patients with histologically confirmed ICC who underwent curative hepatectomy in the First Affiliated Hospital of Sun Yat-sen University (FAH-SYSU) from January 2012 to January 2022 and Sun Yat-sen University Cancer Center (SYSUCC) from January 2018 to January 2022. Patients were excluded if they had positive surgical margin (R1/R2), positive lymph node, received other preoperative anticancer therapy, were presence of distant metastases, combination of other malignancies, or perioperative mortality (within 30 postoperative days). All patients included in the two centers underwent curative-intent hepatectomy. The operative strategies were determined based on the multidisciplinary tumor meeting discussions and performed according to standard clinical practice. Specifically, the extent of surgical resection was considered based on the size, location, and number of the tumor and the presence of cirrhosis. R0 resection was achieved in the presence of sufficient volume of future liver remnant. The extent of lymph node dissection was based on preoperative imaging suspicion of lymph node metastasis or intraoperative evaluation. Remove direct invasion of adjacent structures and local extrahepatic metastases identified intraoperatively. The ethics committees of both participating institutions approved the study. A waiver for informed consent was obtained and patient identifying information was removed.

The prognosis model was developed in the derivation cohort of FAH-SYSU and tested in the external validation cohort of SYSUCC. All histopathologically confirmed ICC were positive for CK7, CK19 and MUC1, and negative for CK20, HepPar1 and glypican-3 (18). The definition of ESICC refers to ICC patients with R0 and lymph node-negative. Considering that compared with R1 and lymph node-positive, ICC patients with R0 and lymph node-negative tend to have a better prognosis, and guidelines do not mandatorily recommend postoperative chemotherapy for such patients (3, 5, 9, 19).

### Data collection

Information was collected according to a standardized datasheet. Demographic and clinicopathological variables included age, gender, medical history (i.e., diabetes, cholelithiasis, hepatitis and cirrhosis), preoperative laboratory values (i.e., neutrophil–lymphocyte ratio [NLR], platelet–lymphocyte ratio [PLR], albumin [ALB], total bilirubin [TBIL], alanine aminotransferase [ALT], aspartate aminotransferase [AST], *γ*-glutamyl transferase [*γ*-GGT], alkaline phosphatase [ALP], carbohydrate antigen 19-9 [CA19-9], carcinoembryonic antigen [CEA]), and tumor-related characteristics (ie tumor number, tumor size, lymph node status, surgical margin width, margin status, tumor differentiation grade, vascular and adjacent organs invasion). Tumor stage was categorized according to the 8th edition of the American Joint Committee on Cancer (AJCC) staging manual (20).

### Follow up

After liver resection, patients were regularly followed and monitored for recurrence by detection of CA19-9, CEA, and imaging scanning including ultrasonography, computed tomography, or/and magnetic resonance imaging, once every 3–6 months for the first two years and once every 6–12 months thereafter. ICC recurrence was defined as suspicious or positive findings on two types of surveillance imaging or histologically confirmed disease. The date of the recurrence was confirmed by reviewing imaging reports, pathology reports, and hospitalization records from the electronic medical records. OS was defined from the date of surgery to the date of death or last follow-up. RFS was measured from the date of surgery to the date of first recurrence or death or last follow-up.

### Statistical analysis

Categorical variables were presented as frequencies with percentages. Continuous variables were presented as means with standard deviations (SD) or medians with interquartile ranges (IQR). Differences between continuous variables were assessed with the Student's *t* tests or Mann–Whitney tests, and differences between categorical variables were assessed using the Fisher's exact tests. In the derivation cohort, the association of clinicopathological variables with the prognosis following ESICC resection was assessed by Cox proportional hazards regression analysis. Variables significant on univariate analysis (*P* < 0.1) were subsequently included into the multivariable Cox regression model, and a step-wise backward selection approach was used to remove nonsignificant variables using a *P* value < 0.05. The risk scores to predict ESICC prognosis were developed based on the *β*-coefficients of the clinicopathological variables significant in the final step of the multivariable Cox regression analysis. Relative risk was expressed as hazard ratios (HR) with 95% confidence interval (CI). The Harrell's concordance index (C-index) was calculated to evaluate the discriminatory performance of the prognosis model. The prognostic discrimination of the model was subsequently estimated in the external validation cohort. Nomogram for 1-, 2-, and 3-year OS probabilities was constructed based on the prognosis model. Moreover, we evaluated the calibration of the nomogram to compare the risk predicted by the nomogram with the observed risk. Receiver operating characteristic curves (ROC) were used to compare the predictive performance of the prognosis model with other prognostic factors for 3-year OS. The median of risk scores was used to classify patients into high- and low-risk groups. Kaplan-Meier curves and log-rank tests were performed to assess the distinctions in OS and RFS between different risk stratifications, and to compare the prognosis model with the preexisting staging system. In addition, the clinical effectiveness and utility were assessed by decision curve analysis (DCA) and net reclassification index (NRI), respectively. All *P* values were two-sided, and *P* < 0.05 was considered statistically significant. All statistical analyses were constructed with Stata/MP version 14.0, and R version 3.6.2.

## Results

### Demographic and clinicopathological variables

A total of 263 patients matched the inclusion and exclusion criteria and were enrolled in the final analytic cohorts, comprising 180 patients in the derivation cohort and 83 patients in the external validation cohort. The demographic and clinicopathological variables are detailed in Table 1. The mean age of patients was 58.0 years (SD, 10.5 years), and 153 patients (58.2%) were male. Diabetes, cholelithiasis, hepatitis, and cirrhosis were present in 41 (15.6%), 50 (19.0%), 108 (41.1%), and 24 (9.1%) patients, respectively. A subset of patients (*n* = 53, 20.2%) were suspicious or positive regional lymph node metastasis on preoperative imaging. Tumor size was universally large, with a median diameter of 5 cm (IQR, 3.5–7.0 cm), and single nodule was predominant (*n* = 205, 77.9%). Most patients underwent wide margin hepatectomy (*n* = 187, 71.1%). Nearly half of the patients held poorly differentiated tumors (*n* = 124, 47.1%), only a few patients had vascular invasion (*n* = 36, 13.7%), and the majority of patients were in the AJCC T1 stage (*n* = 166, 63.1%).

**Table 1:** Demographic and clinicopathological variables of patients.

Variables	Total (*n* = 263)	Derivation cohort (*n* = 180)	Validation cohort (*n* = 83)	*P* value
	No. (%)			
Age, years, mean (SD)	58.0 (10.5)	58.4 (10.3)	57.2 (11.1)	0.404
Gender				0.788
Male	153 (58.2)	106 (58.9)	47 (56.6)	
Female	110 (41.8)	74 (41.1)	36 (43.4)	
Diabetes				1.000
No	222 (84.4)	152 (84.4)	70 (84.3)	
Yes	41 (15.6)	28 (15.6)	13 (15.7)	
Cholelithiasis				0.027
No	213 (81.0)	139 (77.2)	74 (89.2)	
Yes	50 (19.0)	41 (22.8)	9 (10.8)	
Hepatitis				0.022
No	155 (58.9)	115 (63.9)	40 (48.2)	
Yes	108 (41.1)	65 (36.1)	43 (51.8)	
Cirrhosis				<0.001
No	239 (90.9)	173 (96.1)	66 (79.5)	
Yes	24 (9.1)	7 (3.9)	17 (20.5)	
CA19-9, U/ml, median (IQR)	40.2 (10.8–302.6)	49.3 (10.6–518.7)	26.0 (11.2–93.3)	0.024
CEA, μg/L, median (IQR)	2.8 (1.8–5.3)	2.8 (1.9–5.6)	2.7 (1.7–4.1)	0.176
ALB, g/L, median (IQR)	41.5 (37.8–44.7)	39.9 (36.7–42.7)	44.7 (42.9–47.5)	<0.001
ALT, U/L, median (IQR)	21.1 (15.0–33.1)	21.0 (14.5–34.0)	22.0 (15.4–32.5)	0.716
AST, U/L, median (IQR)	25.0 (20.0–32.0)	26.0 (20.0–33.0)	21.5 (18.6–29.9)	0.007
TBIL, μmol/L, median (IQR)	11.8 (9.5–16.1)	12.1 (10.0–16.5)	11.3 (8.7–15.3)	0.029
*γ*-GGT, U/L, median (IQR)	56.0 (33.9–101.6)	61.5 (37.5–123.0)	43.6 (29.0–74.2)	0.002
ALP, U/L, median (IQR)	87.5 (70.8–125.0)	90.5 (76.0–129.5)	81.0 (63.1–105.6)	0.017
NLR, median (IQR)	2.4 (1.8–3.3)	2.6 (1.8–3.4)	2.3 (1.7–3.1)	0.104
PLR, median (IQR)	126.5 (98.7–165.8)	124.2 (95.4–164.5)	132.7 (106.2–167.7)	0.200
LNM on imaging				1.000
Negative	210 (79.8)	144 (80.0)	66 (79.5)	
Suspicious or positive	53 (20.2)	36 (20.0)	17 (20.5)	
Tumor number				0.054
Unifocal	205 (77.9)	134 (74.4)	71 (85.5)	
Multifocal	58 (22.1)	46 (25.6)	12 (14.5)	
Tumor size, cm, median (IQR)	5.0 (3.5–7.0)	5.0 (3.6–7.0)	5.0 (3.5–7.0)	0.962
Surgical margin width				0.002
<1 cm	76 (28.9)	41 (22.8)	35 (42.2)	
≥1 cm	187 (71.1)	139 (77.2)	48 (57.8)	
Tumor differentiation				0.001
Poor	124 (47.1)	72 (40.0)	52 (62.7)	
Moderate/good	139 (52.9)	108 (60.0)	31 (37.3)	
Vascular invasion				0.179
No	227 (86.3)	159 (88.3)	68 (81.9)	
Yes	36 (13.7)	21 (11.7)	15 (18.1)	
Number of LN harvested				0.363
<6	223 (84.79)	150 (83.33)	73 (87.95)	
≥6	40 (15.21)	30 (16.67)	10 (12.05)	
T stage				0.682
T1a/T1b	166 (63.1)	112 (62.2)	54 (65.1)	
T2/T3/T4	97 (36.9)	68 (37.8)	29 (34.9)	
Blood loss				0.002
≤300 ml	167 (63.50)	103 (57.22)	64 (77.11)	
>300 ml	96 (36.50)	77 (42.78)	19 (22.89)	
Blood transfusion				<0.001
No	211 (80.23)	134 (74.44)	77 (92.77)	
Yes	52 (19.77)	46 (25.56)	6 (7.23)	

SD, standard deviation; IQR, interquartile range; CA19-9, carbohydrate antigen 19-9; CEA, carcinoembryonic antigen; ALB, albumin; ALT, alanine aminotransferase; AST, aspartate aminotransferase; TBIL, total bilirubin; γ-GGT, γ-glutamyl transferase; ALP, alkaline phosphatase; NLR, neutrophil–lymphocyte ratio; PLR, platelet–lymphocyte ratio; LNM, lymph node metastasis.

### Identification of risk factors for prognosis

After a median follow-up of 42.4 months (95% CI, 32.4–46.6 months) in the derivation cohort, 77 (42.8%) patients recurred within one year after surgery and median OS was 33.8 months (95% CI 27.9–45.6 months). The 3-year OS was 49.0% (95% CI, 40.0–57.4%). Differential coefficients of 13 clinicopathological covariates were associated with outcomes in univariate Cox regression analysis of the derivation cohort (Table 2, Supplementary Figure S1). Among them, five factors showed significant in a further multivariable analysis, which demonstrated that higher count of CA19-9 (HR, 1.01; 95% CI, 1.00–1.01) and CEA (HR, 1.01; 95% CI, 1.00–1.01), larger tumor size (HR, 1.15; 95% CI, 1.07–1.23), and T2–T4 stage disease (HR, 1.78; 95% CI, 1.15–2.78) were respectively associated with lower odds of survival, whereas well or moderately differentiated tumors (HR, 0.54; 95% CI, 0.34–0.85) were again associated with greater odds of survival (Table 2, Supplementary Figure S2). None of the remaining variables noted to be independent predictors of survival.

**Table 2:** Univariate and multivariable Cox regression analysis of factors associated with survival in the derivation cohort.

Variables	Univariate analysis		Multivariable analysis	
	HR (95% CI)	*P* value	HR (95% CI)	*P* value
Age (≥60 years vs. <60 years)	1.14 (0.74–1.75)	0.562		
Gender (male vs. female)	1.01 (0.66–1.55)	0.967		
Diabetes (yes vs. no)	1.20 (0.68–2.14)	0.531		
Cholelithiasis (yes vs. no)	1.47 (0.90–2.42)	0.125		
Hepatitis (yes vs. no)	0.92 (0.59–1.43)	0.698		
Cirrhosis (yes vs. no)	0.95 (0.38–2.36)	0.912		
CA19-9, every 100-U/ml increase	1.01 (1.00–1.02)	**0**.**001**	1.01 (1.00–1.01)	**0**.**030**
CEA	1.01 (1.00–1.01)	**0**.**001**	1.01 (1.00–1.01)	**0**.**005**
ALB	0.95 (0.91–1.00)	**0**.**039**		
ALT	1.00 (1.00–1.01)	**0**.**033**		
AST	1.00 (1.00–1.01)	**0**.**092**		
TBIL	1.00 (0.99–1.00)	0.252		
γ-GGT	1.00 (1.00–1.00)	**0**.**044**		
ALP	1.00 (1.00–1.00)	**0**.**011**		
NLR	1.33 (1.16–1.54)	**<0.001**		
PLR	1.00 (1.00–1.01)	**0**.**012**		
LNM on imaging (suspicious or positive vs. negative)	1.50 (0.89–2.54)	0.125		
Tumor number (multifocal vs. unifocal)	1.75 (1.11–2.77)	**0**.**015**		
Tumor size	1.17 (1.10–1.25)	**<0.001**	1.15 (1.07–1.23)	**<0.001**
Surgical margin width (≥1 cm vs. <1 cm)	0.85 (0.50–1.43)	0.535		** **
Tumor differentiation (moderate/good vs. poor)	0.58 (0.37–0.90)	**0**.**016**	0.54 (0.34–0.85)	**0.007**
Vascular invasion (yes vs. no)	1.22 (0.61–2.46)	0.574		** **
Number of LN harvested (≥6 vs. <6)	0.88 (0.48–1.61)	0.671		** **
T stage (T2/T3/T4 vs. T1a/T1b)	1.93 (1.25–2.97)	**0**.**003**	1.78 (1.15–2.78)	**0.010**
Blood loss (>300 ml vs. ≤300 ml)	1.43 (0.93–2.19)	0.103		** **
Blood transfusion (Yes vs. No)	1.86 (1.18, 2.91)	0.007		** **

Statistically significant *P* values are in bold.

HR, hazard ratio; CI, confidence interval; CA19-9, carbohydrate antigen 19-9; CEA, carcinoembryonic antigen; ALB, albumin; ALT, alanine aminotransferase; AST, aspartate aminotransferase; TBIL, total bilirubin; γ-GGT, γ-glutamyl transferase; ALP, alkaline phosphatase; NLR, neutrophil–lymphocyte ratio; PLR, platelet–lymphocyte ratio; LNM, lymph node metastasis.

### Development of prognosis model

**Figure 1:**
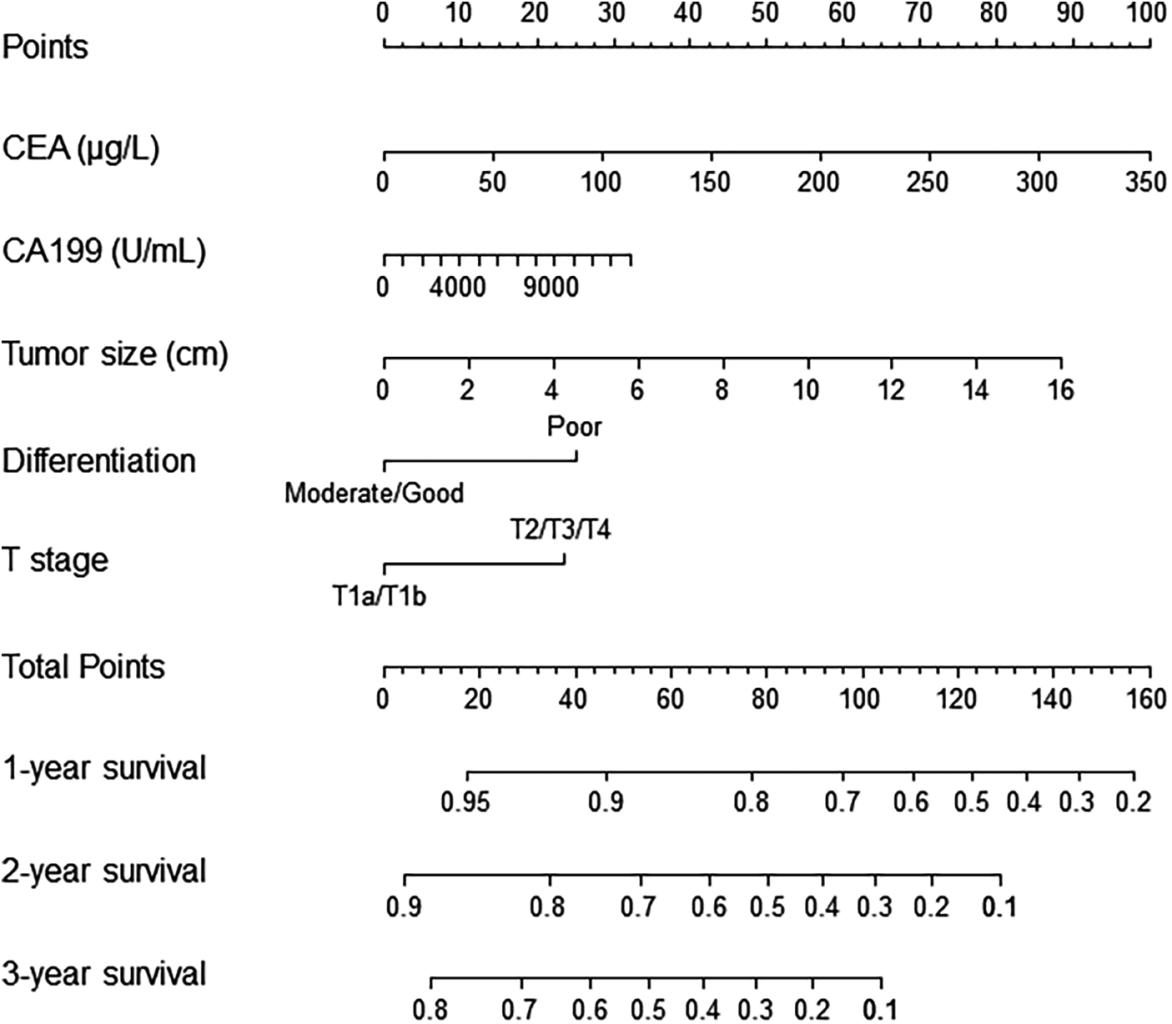
Nomogram for prediction of survival in patients with ESICC after resection. The independent prognostic factors from the multivariable Cox regression analysis were located in each variable axis, and a line is drawn up to the Points axis to determine the corresponding points for each variable. The sum of these points is located in the Total Points axis, and a line is drawn down to the survival axes to determine the 1-, 2- or 3-year survival probability. CA19-9, preoperative serum CA19-9; CEA, preoperative serum carcinoembryonic antigen.

**Figure 2:**
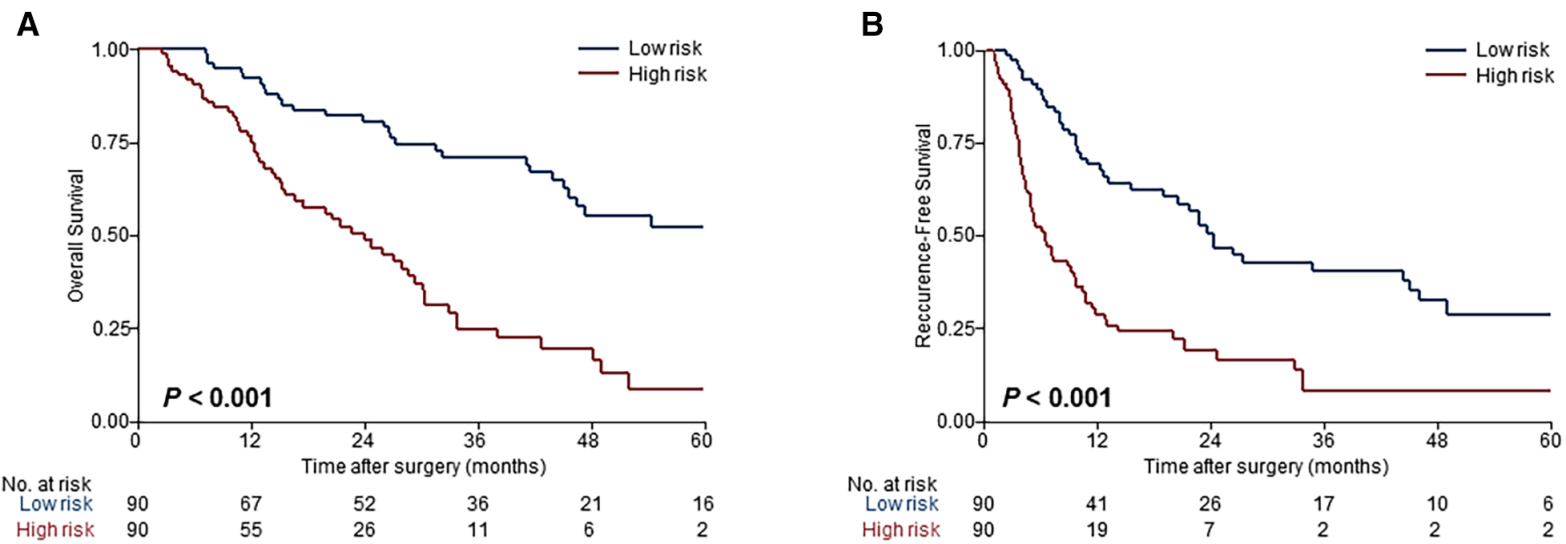
Evaluation of the model performance for risk-dependent stratification in the derivation cohort. Kaplan-Meier curves demonstrated differences in overall survival (**A**) and recurrence-free survival (**B**) between low- and high-risk patients with ESICC based on the prognosis model.

Risk scores were developed based on the aforementioned five independent prognostic factors and a nomogram was constructed to estimate 1-, 2-, and 3-year survival probability (Figure 1). The discriminatory performance of the prognosis model was satisfying in the derivation cohort (C-index, 0.71; 95% CI, 0.65–0.77), and superior to the discriminative accuracy of the 8th edition AJCC TNM staging system, which had a C-index of 0.59 (95% CI, 0.53–0.65). Meanwhile, the prognosis model outperformed individual prognostic parameters, including CA19-9 (C-index, 0.64; 95% CI, 0.56–0.71), CEA (C-index, 0.59; 95% CI, 0.52–0.65), tumor size (C-index, 0.66; 95% CI, 0.60–0.73), tumor differentiation (C-index, 0.57; 95% CI, 0.52–0.63) and T stage (C-index, 0.57; 95% CI, 0.51–0.63) (Supplementary Figure S3A). Furthermore, the calibration cures indicated overall good agreement between the predicted and the observed probabilities of OS in the prognosis model (Supplementary Figure S3B). The NRI further demonstrated that the model had higher predictive power than the TNM staging system and other prognostic markers (Supplementary Table S1). In addition, the DCA showed that using this prognosis model to predict survival provided more clinical benefit than TNM system (Supplementary Figure S3C).Based on the risk scores, patients were noticeably stratified into high — and low-risk groups. The survival of low-risk patients was significantly better than that of high-risk patients, with 3-year OS of 70.9% and 24.9% (*P *< 0.001), respectively (Figure 2A). Moreover, the model accurately distinguished RFS between high- and low-risk patients, with the low-risk subgroup having a longer RFS compared to the high-risk subgroup (median RFS: low risk, 24.3 months vs. high risk, 6.4 months; *P *< 0.001) (Figure 2B).

### External validation of the prediction model

The clinicopathological characteristics of the patients in the external validation cohort are shown in Table 1. In this cohort, the median OS was 49.5 months (95% CI, 31.5-NA months), the number of recurrences within one year was 26 (31.3%), and the 3-year OS was 69.6% (95% CI, 52.8–81.4%). Validation of the proposed model was performed in this independent cohort from another clinical center. In the assessment of discriminative capacity, the prognosis model exhibited remarkable accuracy (C-index, 0.78; 95% CI, 0.67–0.88) overperforming the 8th edition AJCC staging system (C-index, 0.54; 95% CI, 0.41–0.67) and the individual prognostic parameters (CA19-9 [C-index, 0.65; 95% CI, 0.47–0.82), CEA [C-index, 0.62; 95% CI, 0.46–0.78], tumor size [C-index, 0.60; 95% CI, 0.46–0.74], tumor differentiation [C-index, 0.57; 95% CI, 0.46–0.69] and T stage [C-index, 0.54; 95% CI, 0.41–0.67]) (Supplementary Figure S4A), with a good calibration (Supplementary Figure S4B). Compared to the TNM staging system and other prognostic variables, the model had higher NRI values (Supplementary Table S1) and net clinical benefit (Supplementary Figure S4C).

**Figure 3:**
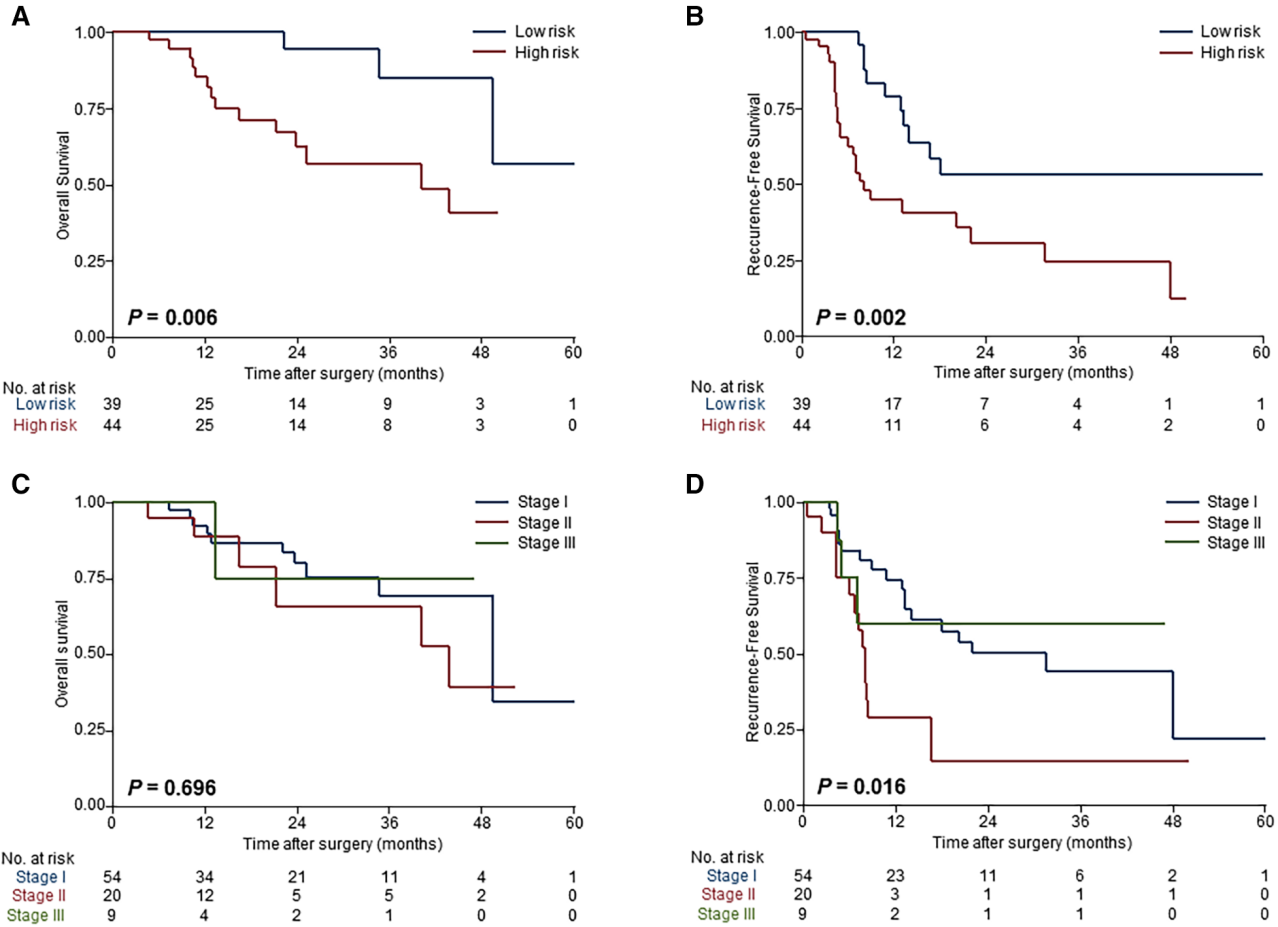
Validation of the model performance for risk-dependent stratification in the external validation cohort. Kaplan-Meier curves demonstrated significant differences in overall survival (**A**) and recurrence-free survival (**B**) between low-risk and high-risk patients with ESICC according to the prognostic model. Risk stratification based on the TNM staging system showed that there were no differences among patients with different risk groups in overall survival (**C**) and recurrence-free survival (**D**).

Our intent of categorization was achieved excellently in the validation cohort that risk stratifications based on the prognosis model showed homogeny to the derivation cohort (Figures 3A,B), and superior risk-dependent OS and RFS observed in the prognosis model compared to the TNM staging system (Figures 3C,D). Specifically, patients deemed high risk had a worse OS and RFS compared with patients who were low risk. The detailed 3-year OS, RFS at 6 months and 1 year, and hazard ratios for both high- and low-risk groups are shown in Table 3.

**Table 3:** Risk-dependent survival according to the prognosis model.

Grade	Derivation cohort					External validation cohort				
	3-year OS (%)	HR (95% CI)	6-month RFS (%)	1-year RFS (%)	HR (95% CI)	3-year OS (%)	HR (95% CI)	6-month RFS (%)	1-year RFS (%)	HR (95% CI)
Low risk	70.9	1.00	89.2	69.2	1.00	85.0	1.00	100.0	78.7	1.00
High risk	24.9	3.71 (2.32–5.96)	52.5	28.8	2.97 (1.98–4.46)	56.9	5.01 (1.42–17.64)	65.3	44.9	2.95 (1.42–6.15)

OS, overall survival; RFS, recurrence-free survival; HR, hazard ratio.

## Discussion

In this study, we sought to develop a predictive prognosis model for ESICC after resection using a multivariable Cox proportional hazards regression model that incorporated clinical and pathological variables, such as CA19-9, CEA, tumor size, tumor differentiation grade, and T stage. This is the first prognosis model for patients with ESICC after curative resection. This model was approved to have outstanding discrimination power for survival outcomes with a C-index of 0.71 in the derivation cohort and robust external validation in another independent cohort (C-index, 0.78). The performance was superior to the 8th edition AJCC TNM staging system and well calibrated. The prognosis model can accurately identify high-risk patients who are prone to recurrence and have poor survival, which is helpful to guide personalized postoperative cancer care.

Lymph node metastasis and positive surgical margin are previously recognized as significant unfavorable prognostic factors for ICC patients after curative hepatectomy (21). However, patients with R0 resection and node-negative, namely ESICC, still experience recurrence (4). At present, the TNM staging system does not incorporate clinical and pathological variables to predict RFS and OS, and for ESICC patients only the T stage can be referred to assess risk grades (20). Whereas, in clinical practice, tumor survival risk prediction based on a single risk factor is often insufficient to rationalize postoperative treatment (22). In addition, the guidelines for postoperative surveillance recommend imaging every 3–6 months for 2 years, and then every 6–12 months for up to 5 years (9). Unfortunately, imaging surveillance has a low medical follow-up and low accuracy in detecting early recurrence (23). Postoperative monitoring of ESICC patients with high recurrence characteristics based on this criterion may be inadequate. It hints that the current staging system and guidelines are ineligible to perform precise estimation of outcomes of ESICC after resection.

Our present study is an essential step forward in this regard, as it fills a gap in the current lack of means to identify recurrence or early death in ESICC following complete surgical resection. The prognosis model constructed in this study incorporated five prognostic determinants, namely CA19-9, CEA, tumor size, tumor differentiation grade, and T stage, which were previously confirmed prognostic factors (24–27). In addition, in our prognosis model, tumor size is a continuous variable. In contrast, tumor size in the T stage is a two-dimensional variable that cannot quantify the specific negative prognostic impact of each unit increase in tumor size. Moreover, the T stage also includes information on the tumor number, vascular invasion, local extent, and adjacent structure involvement. The inclusion of both tumor size and T stage allows for a more comprehensive assessment. Overall, the data highlight how patients with high CA19-9 and CEA, large tumor size, poor differentiation, and low T stage have a markedly higher likelihood of experiencing poor survival. These patients are more likely to benefit from adjuvant therapy and more rigorous cancer surveillance.

According to current clinical practice guidelines, postsurgical treatment options for ESICC include observation alone, adjuvant chemo (radio) therapy, and clinical trials (5, 9, 19). Additionally, the era of precision medicine addressed targeted therapy and immunotherapy based on individually unique genetic alterations and immune profiles, which have the potential to be therapeutic approaches to improve patient prognosis (28, 29). There is not yet convincing enough evidence to make appropriate treatment choices for ESICC patients (30). Importantly, the possibility of tumor recurrence remains one of the most prominent risk factors in determining which subset of patients should receive postoperative adjuvant therapy. Furthermore, there should be a cautious trade-off between the disadvantages and survival benefits of adjuvant therapy (31). The prognosis model developed in this study can accurately identify high-risk patients of ESICC, thus providing patients with individual treatment decisions including increased appropriate use of adjuvant therapy for high-risk patients to prolong relapse-free time. Or conversely, for low-risk ESICC with no other indications for treatment, the toxicity and additional expense of adjuvant therapy can be waived.

Our report is unique for the following reasons. First, our study focuses only on the ESICC rather than all periods of ICC. A staged analysis of patients is more conducive to precise individualized treatment. Second, the prognosis model contains clinicopathological variables such as CA19-9, CEA, tumor size, tumor differentiation, and T-stage, which can be universally available among medical centers. There are several potential limitations of our study. First, our retrospective study design might lead to potential selection biases. In addition, ESICC excludes N1 and M1 patients, which may be biased when compared with the TNM staging system. Hence, prospective clinical trials are desired to validate the accuracy of our model. Second, the sample size of ESICC is limited by the low incidence and high progression rate of ICC, which could also be the reason why there was no statistical difference in the validation cohort, although the model had a higher NRI than CA19-9. In the subsequent work, we will further validate the efficacy of the prognosis model on a larger prospective sample. Third, the model data was obtained from two medical institutions, patient selection and surgical techniques may vary across participating centers depending on local practices. Although the model performed very well in the external validation cohort, additional medical centers are needed to validate the generalizability of this model.

In summary, we developed a prognosis model based on clinical and pathological variables for predicting the survival outcomes of ESICC patients after radical surgery. Risk stratification based on the model may be a practical tool to identify prognosis risk of ESICC, which can provide valuable clinical suggestions for the approaches of adjuvant therapy and the frequency of postoperative surveillance. Prospective multi-institutional studies are needed to further validate this predictive prognosis model.

## Data Availability

The original contributions presented in the study are included in the article/**Supplementary Material**, further inquiries can be directed to the corresponding author/s.
