# Supplementary material for: Development and external validation of a prognosis model to predict outcomes after curative resection of early-stage intrahepatic cholangiocarcinoma
Source: Front Surg. 2023 Mar 8;10:1102871. doi: 10.3389/fsurg.2023.1102871 (PMC10030709; doi:10.3389/fsurg.2023.1102871)
Supplement: Supplementary file 1 [file Table1.docx]

**Supplementary Table S1.** NRI of the prognosis model, TNM staging system, and other prognostic markers.

|  | Derivation cohort | | Validation cohort | |
| --- | --- | --- | --- | --- |
| Prognosis model | NRI | *P* value | NRI | *P* value |
| vs CA19-9 | 0.38 | 0.014 | 0.10 | 0.669 |
| vs CEA | 0.54 | < 0.001 | 0.46 | 0.024 |
| vs Tumor size | 0.21 | 0.122 | 0.69 | 0.003 |
| vs Tumor differentiation | 0.59 | < 0.001 | 1.09 | < 0.001 |
| vs T stage | 0.59 | < 0.001 | 0.73 | 0.002 |
| vs TNM | 0.41 | 0.004 | 0.87 | 0.001 |

NRI, net reclassification index; OS, overall survival; CA19-9, carbohydrate antigen 19-9; CEA, carcinoembryonic antigen.
