# Supplementary figures and images for: Development and external validation of a prognosis model to predict outcomes after curative resection of early-stage intrahepatic cholangiocarcinoma
Source: Front Surg. 2023 Mar 8;10:1102871. doi: 10.3389/fsurg.2023.1102871 (PMC10030709; doi:10.3389/fsurg.2023.1102871)

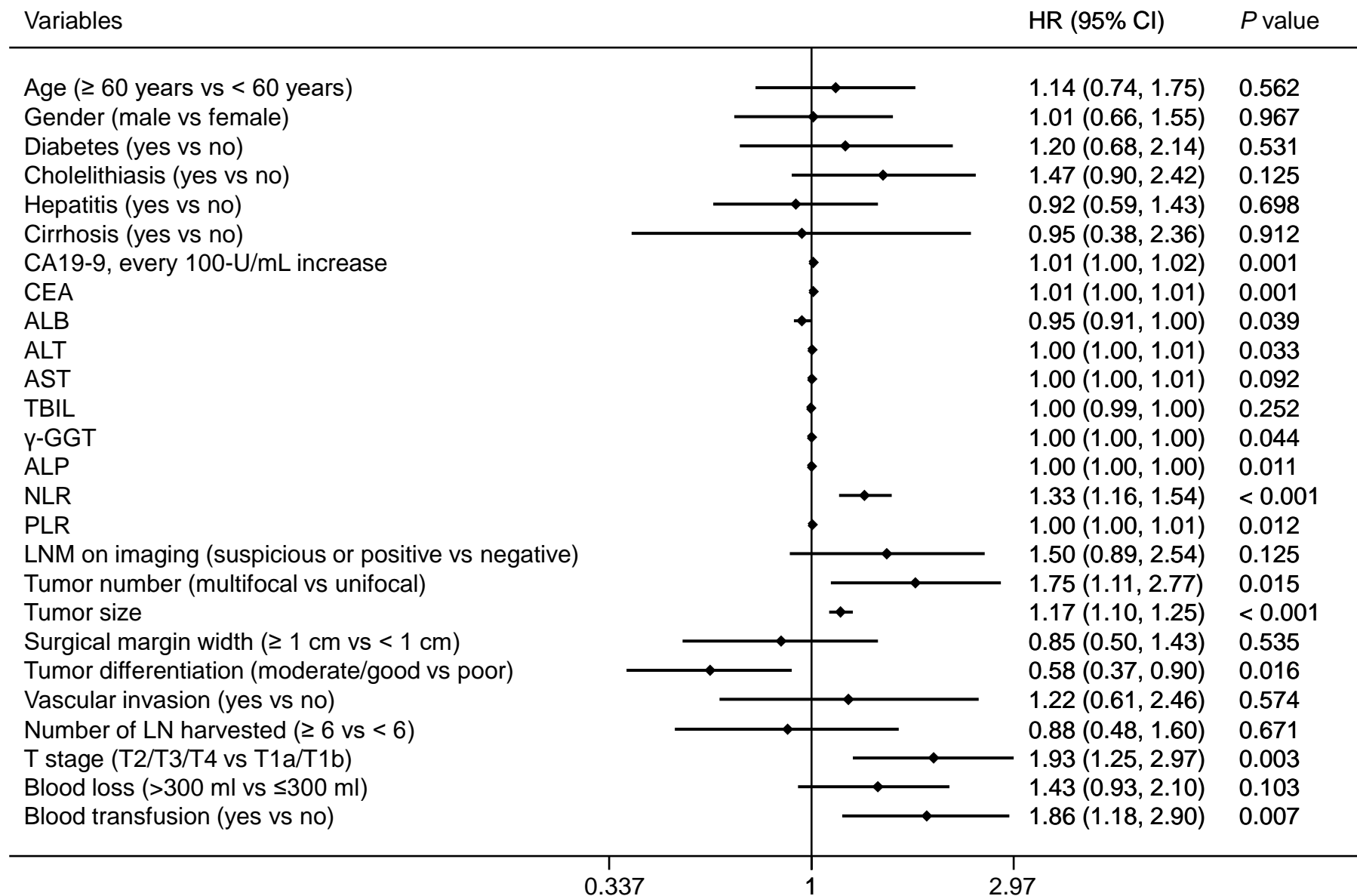

Supplement: Supplementary file 2 [file Image1.pdf]

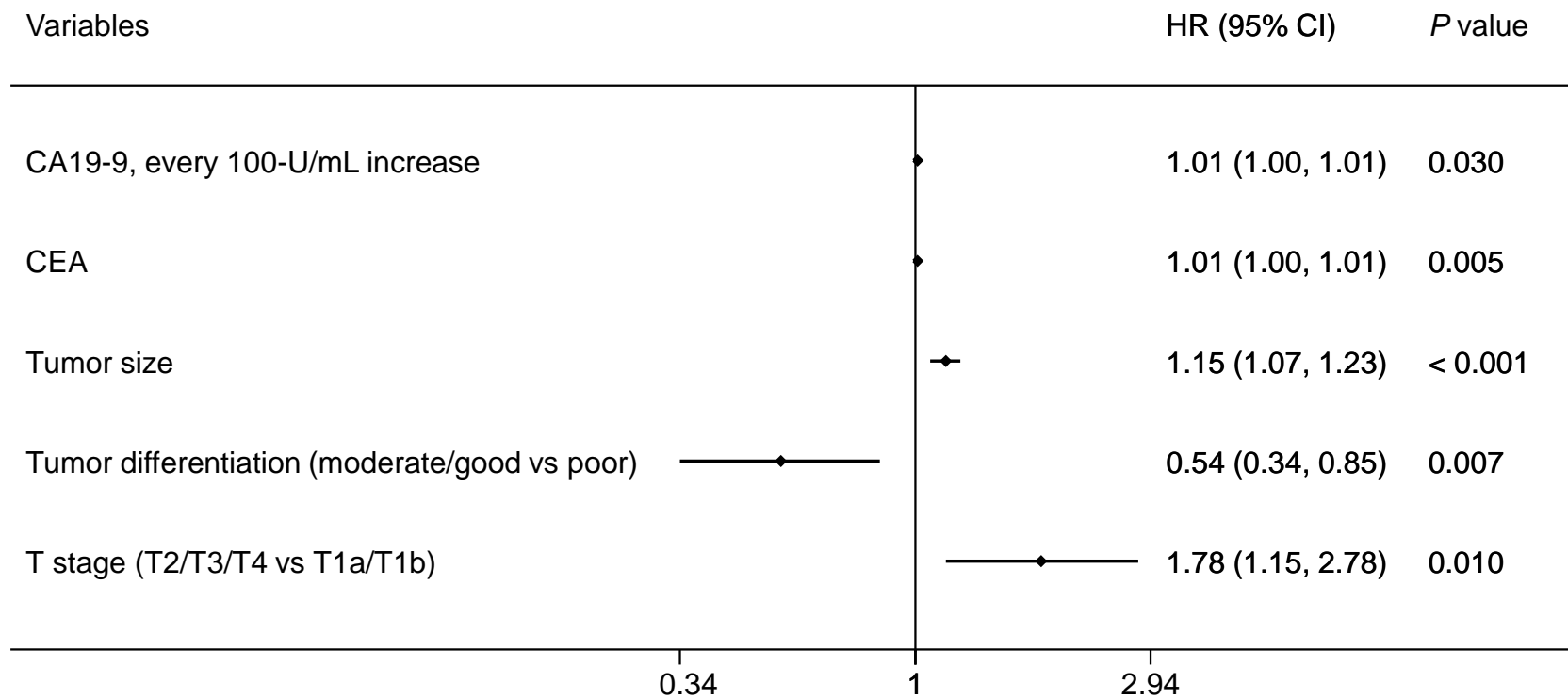

Supplement: Supplementary file 3 [file Image2.pdf]

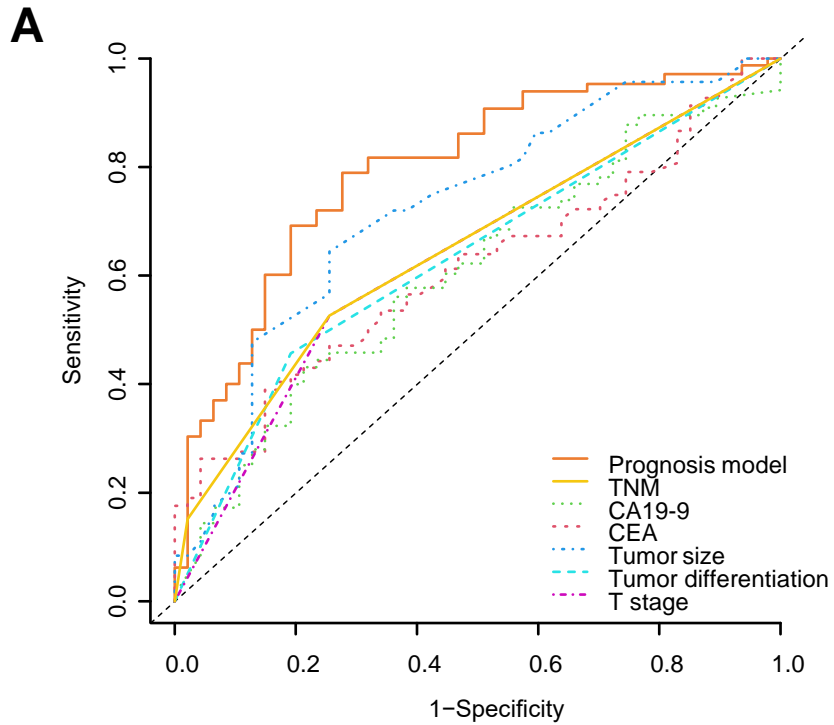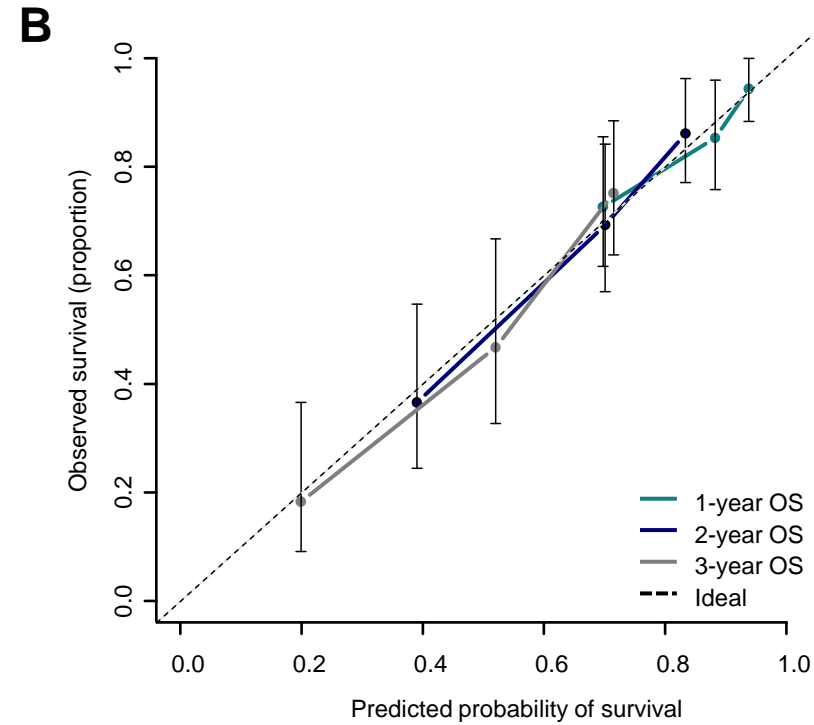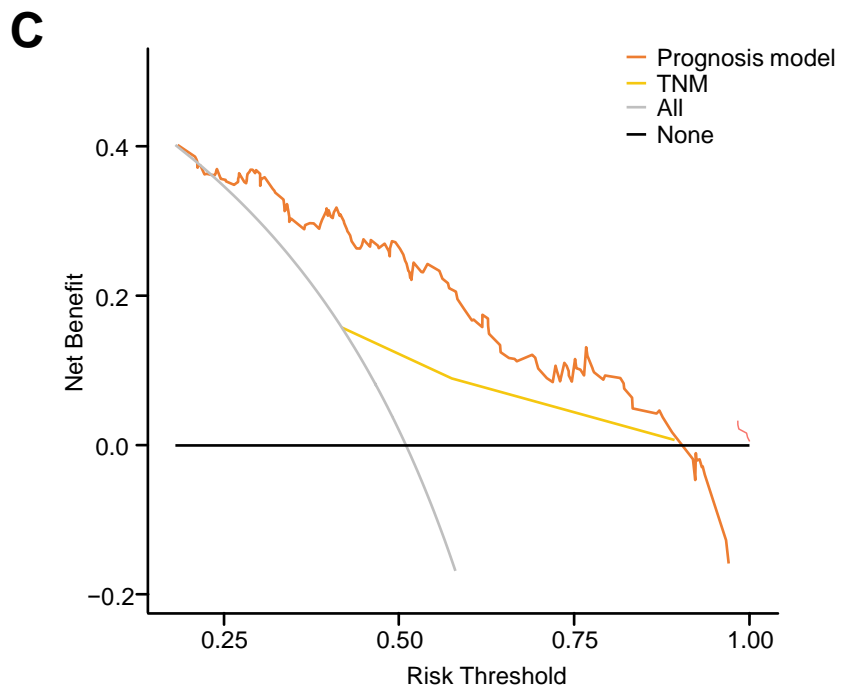

Supplement: Supplementary file 4 [file Image3.pdf]

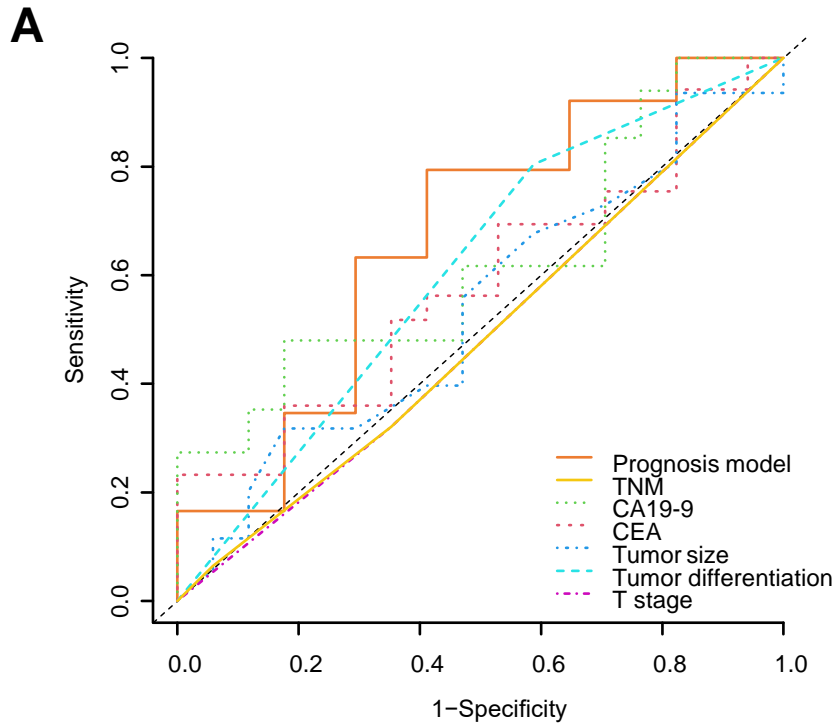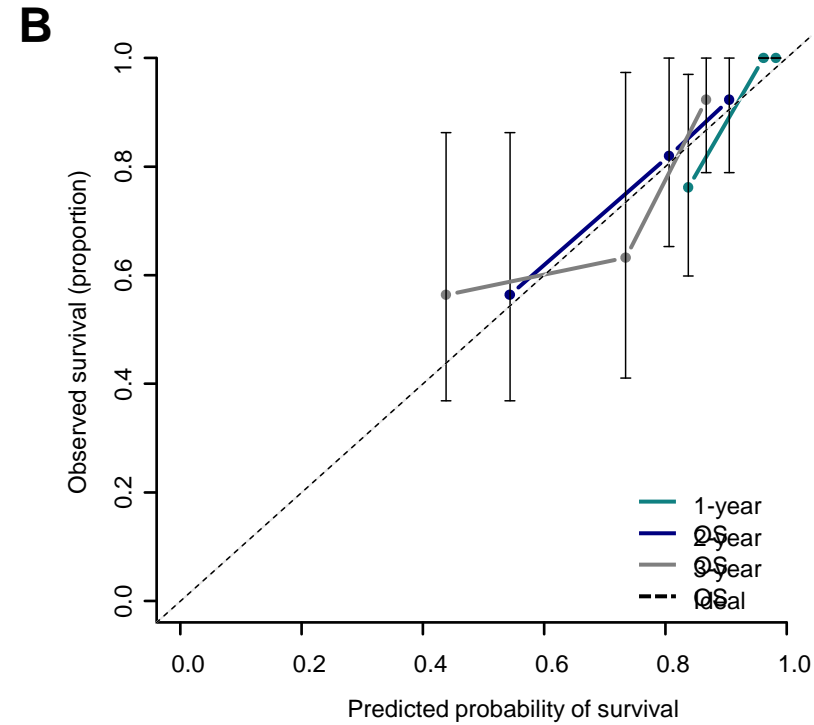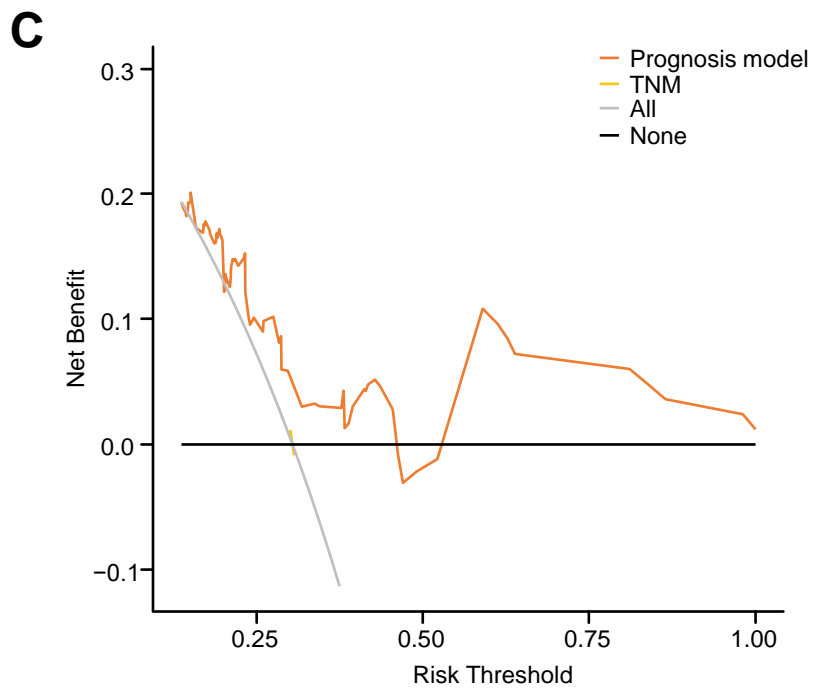

Supplement: Supplementary file 5 [file Image4.pdf]
